# Retrospective evaluation of ERAS-guided rehabilitation nursing in anterior cervical discectomy and fusion patients

**DOI:** 10.3389/fmed.2025.1657725

**Published:** 2025-10-01

**Authors:** Guilin Liu, Juan Xiao, Siqi Wei, Lishi Pang, Chunfeng Xing

**Affiliations:** ^1^Department of Spinal Orthopedics, Shenzhen Guangming; ^2^Department of Nursing, Shenzhen Guangming District People’s Hospital, Shenzhen, Guangdong, China

**Keywords:** anterior cervical discectomy and fusion (ACDF), enhanced recovery after surgery (ERAS), gastrointestinal function, pain management, spine surgery

## Abstract

**Purpose:**

This study aimed to evaluate the efficacy of an ERAS-based perioperative nursing pathway in patients undergoing anterior cervical discectomy and fusion (ACDF).

**Methods:**

A retrospective analysis was conducted on 156 ACDF patients (2022–2024), with 80 in the ERAS group and 76 in the control group. The ERAS group received interdisciplinary care including preoperative nutritional optimization, intraoperative hemodynamic stabilization, early mobilization, and multimodal analgesia. Primary endpoints: time to first flatus and length of hospital stay. Secondary endpoints: time to first defecation, time to first solid food intake, VAS pain scores (resting/moving), urinary catheter removal time, first ambulation time, JOA, NDI, PONV, and complications. Statistical analyses compared outcomes between groups using *t*-tests and chi-squared tests.

**Results:**

The ERAS group demonstrated significantly shorter times to first flatus (7.89 ± 2.35 vs. 12.56 ± 4.12 h, *P* < 0.001), defecation (15.92 ± 5.34 vs. 22.89 ± 7.23 h, *P* < 0.001), and solid food intake (8.87 ± 2.42 vs. 13.58 ± 3.56 h, *P* < 0.001). Resting and dynamic VAS scores were lower in the ERAS group (*P* < 0.001 for both), with shorter urinary catheter removal time (8.36 ± 2.01 vs. 14.25 ± 3.72 h, *P* < 0.001), first ambulation time (9.27 ± 2.34 vs. 15.34 ± 4.18 days, *P* < 0.001), and hospital stay (6.35 ± 1.89 vs. 8.12 ± 2.15 days, *P* = 0.002). At 3 months, the ERAS group showed better JOA scores (16.12 ± 1.03 vs. 15.33 ± 0.98, *P* < 0.05) and lower NDI (8.96 ± 1.32 vs. 10.15 ± 0.60, *P* < 0.05). Complications (e.g., dysphagia, infection) did not differ significantly between groups (*P* = 0.221).

**Conclusion:**

Enhanced recovery after surgery-guided nursing improves gastrointestinal function, reduces pain, and accelerates functional recovery in ACDF patients without increasing complications. This interdisciplinary approach enhances perioperative care efficiency and supports patient-centered outcomes.

## 1 Introduction

Cervical spondylosis, a prevalent spinal disorder characterized by intervertebral disk degeneration and secondary pathological changes, manifests in a spectrum of clinical syndromes such as cervical spondylotic myelopathy and radiculopathy. Patients often present with dizziness, headache, nausea, blurred vision, palpitations, limb numbness, and motor dysfunction, all of which significantly impair quality of life (1). When conservative treatments fail, anterior cervical discectomy and fusion (ACDF) has emerged as the preferred surgical intervention for relieving neural compression and restoring cervical stability (2). Despite its efficacy in addressing spinal cord or root compression, ACDF is associated with postoperative challenges, including neck-shoulder pain, limb numbness, and delayed functional recovery (3, 4). The procedure requires removal of degenerated disks and insertion of fusion materials to decompress the spinal canal and stabilize the cervical spine (5). However, its proximity to critical anatomical structures (e.g., trachea, esophagus, and neurovascular bundles) increases the risk of complications such as hoarseness, dysphagia, and delayed gastrointestinal recovery (6). Moreover, postoperative pain and restricted mobility contribute to prolonged hospitalization and suboptimal functional outcomes (7), while long-term issues such as accelerated degeneration of adjacent segments may further compromise patient prognosis (8, 9). These challenges highlight the need for optimized perioperative management strategies tailored to ACDF.

Enhanced recovery after surgery (ERAS), first proposed by Kehlet (10), advocates evidence-based perioperative interventions aimed at minimizing surgical stress, reducing complications, and accelerating recovery (10, 11). This multidisciplinary model integrates preoperative optimization, intraoperative precision, and postoperative rehabilitation to improve clinical outcomes. ERAS has demonstrated benefits across surgical fields including orthopedics and general surgery. (12) In spinal fusion procedures, ERAS-based nutritional and rehabilitation interventions have been shown to improve wound healing, reduce opioid use, shorten hospital stay, and accelerate functional recovery (13, 14). However, evidence specific to cervical spine surgery remains limited, and the anatomical and functional complexities of the cervical region may necessitate tailored ERAS strategies.

Therefore, this study aims to evaluate the efficacy of an ERAS-guided rehabilitation nursing protocol in ACDF patients. We hypothesize that a structured ERAS protocol–including preoperative nutritional support, intraoperative hemodynamic stabilization, and postoperative early mobilization with multimodal analgesia–will improve gastrointestinal recovery, reduce pain, and shorten hospitalization compared with conventional nursing care. By systematically analyzing outcomes such as time to first flatus, pain scores, and functional recovery indices, this study seeks to establish evidence supporting ERAS integration into cervical spine surgery, ultimately promoting patient-centered care in this population.

## 2 Materials and methods

### 2.1 Patient selection

A retrospective analysis was conducted on 156 patients who underwent anterior cervical discectomy and fusion (ACDF) at Shenzhen Guangming Distract People’s Hospital between December 2022 and December 2024. Of these, 80 patients received perioperative care guided by the enhanced recovery after surgery (ERAS) philosophy (observation group), while 76 patients received conventional perioperative management (control group). The ERAS program for ACDF was introduced in April 2023, following staff training and protocol finalization. From December 2022 to March 2023, all patients received conventional perioperative care; from April 2023 onward, ERAS was progressively implemented across wards. All eligible patients in these windows were screened consecutively.

#### Inclusion criteria:

① First-time ACDF recipients;

② Age ≥ 18 years;

③ Conscious and capable of normal communication.

#### Exclusion criteria:

① Comorbidities involving cardiac, pulmonary, cerebral, or other vital organ dysfunction;

② Malignant tumors;

③ Cognitive or psychiatric disorders.

A total of 22 patients were excluded (8 with prior cervical surgery, 6 with severe cardiopulmonary or renal comorbidities, 3 with malignancies, and 5 with incomplete records or loss to follow-up). The final cohort included 156 patients, of whom 76 received conventional perioperative care and 80 received ERAS-based perioperative care. Group allocation was based on the availability of the care pathway rather than patient preference. The study was approved by the Ethics Committee of Shenzhen Guangming District People’s Hospital (Approval No: LL-KT-2025074), and all participants provided informed consent.

### 2.2 Interventions

#### 2.2.1 Control group (conventional nursing)

Patients received conventional nursing care, including:

Preoperative: Provide standard health education about surgical procedures, covering details like incision location, expected duration, and main steps. Instruct on perioperative precautions such as not wearing jewelry during surgery and following specific skin preparation requirements. Offer postural guidance for early ambulation, demonstrating correct sitting, standing, and initial walking postures to prevent falls and facilitate recovery.

Postoperative: Conduct routine vital sign monitoring, checking body temperature, heart rate, blood pressure, and respiratory rate at regular intervals. Perform wound care, including cleaning, dressing changes following aseptic techniques, and observing for signs of infection like redness, swelling, or discharge. For catheter care, ensure proper fixation, monitor urine output, and follow standard procedures for catheter removal. Use the VAS to assess pain at regular intervals, usually every 2–4 h depending on the patient’s condition. Encourage early mobilization according to standard protocols, which involve helping the patient sit up within a certain time after surgery, followed by standing and short walks, gradually increasing the activity level as tolerated.

#### 2.2.2 Observation group (ERAS-guided care)

An interdisciplinary ERAS team comprising 2 spine surgeons, 2 anesthesiologists, 1 nutritionist, 1 rehabilitation physician, 1 head nurse, and 12 nurses developed and implemented the protocol, as described below:

**Preoperative Phase:**

① Nutritional status was assessed using the Nutritional Risk Screening 2002 (NRS-2002) (15);

② Comprehensive education was provided on surgery, anesthesia, nil per os (NPO) guidelines (6-h fasting for solids, 2-h fasting for clear liquids), postoperative analgesia, early mobilization protocols, and antiemetic strategies to alleviate anxiety;

③ Prehabilitation exercises (e.g., cervical range-of-motion training, swallowing drills) were initiated 2–4 days prior to surgery.

**Intraoperative phase:**

① Hemodynamic stability was maintained with mean arterial pressure and heart rate within ±20% of baseline values;

② Prophylactic antiemetics (ondansetron 4 mg and dexamethasone 5 mg) were administered before anesthesia induction;

③ Normothermia was preserved via controlled room temperature (23 °C–25 °C) and warmed intravenous fluids.

**Postoperative Phase:**

① Mobilization: Patients were assisted to sit at the bedside with a cervical collar 4 h after anesthesia recovery, followed by supervised early ambulation based on surgical tolerance;

② Nutrition: Oral intake was initiated with small volumes of warm water or carbohydrate drinks upon recovery of swallowing reflexes. Non-elderly patients received semi-liquid diets 4 h postoperatively, advancing to regular diets after flatus or defecation. Oral nutritional supplements were prioritized for at-risk patients, with parenteral nutrition reserved for those unable to tolerate enteral intake;

③ Analgesia: Multimodal analgesia included scheduled pregabalin (75 mg orally) and celecoxib (200 mg orally), with tramadol (100 mg intramuscularly) as rescue medication for breakthrough pain;

④ Catheter Management: Urinary catheters were removed early (within 24–48 h) to reduce infection risk.

### 2.3 Outcome measures

Primary endpoints were defined as key markers of perioperative recovery: time to first flatus and length of hospital stay.

Secondary endpoints included multiple dimensions of recovery and safety. Gastrointestinal recovery was assessed by time to first defecation, time to first solid food intake, and incidence of postoperative nausea and vomiting (PONV). Pain and early functional recovery were evaluated using resting and dynamic Visual Analog Scale (VAS) scores (0–10, higher = worse pain), urinary catheter removal time, and first ambulation time. Long-term functional outcomes were measured by Japanese Orthopedic Association (JOA) scores (17-point, higher = better function) and Neck Disability Index (NDI) (0–100%, lower = less disability) at 24 h and 3 months postoperatively. Postoperative complications, including dysphagia, hoarseness, wound infection, and cerebrospinal fluid leakage, were also recorded as secondary endpoints.

### 2.4 Statistical analysis

Statistical analyses were performed using SPSS 20.0 (IBM, USA). Continuous variables are presented as mean ± standard deviation (SD) and compared using Welch’s *t*-test. Categorical variables are expressed as counts and percentages and analyzed using chi-square tests or Fisher’s exact tests. Between-group mean differences and their 95% confidence intervals (95% CI) and *P*-values are reported, with primary endpoints tested at α = 0.05. For secondary endpoints, the false discovery rate (FDR) was controlled using the Benjamini-Hochberg procedure. *P*-values < 0.05 for primary endpoints were considered statistically significant.

## 3 Results

### 3.1 Baseline characteristics of patients

Table 1 presents the baseline demographic and clinical data of the 156 enrolled ACDF patients, who were assigned to the control group (conventional nursing, *n* = 76) or observation group (ERAS-guided care, *n* = 80) based on the perioperative care pathway available during the study period. There were no statistically significant differences between the two groups in terms of sex (*P* = 0.344), number of surgical segments (*P* = 0.208), ASA classification (*P* = 0.556), or preoperative VAS score (*P* = 0.280). Although the observation group had a slightly higher mean age (59.15 ± 10.52 years) than the control group (55.81 ± 11.98 years), the difference did not reach statistical significance (*P* = 0.0602). Data on operative time, intraoperative blood loss, smoking status, and diabetes were not consistently documented in the retrospective dataset and thus were not included in the analysis.

**TABLE 1:** Baseline characteristics of the study cohort.

Characteristics	Control group (*n* = 76)	Observation group (*n* = 80)	χ^2^/t	*P*-value
Age (years), mean ± SD	55.81 ± 11.98	59.15 ± 10.52	1.892*	0.0602
Sex (male/female), *n* (%)	46 (60.5%)/30 (39.5%)	54 (67.5%)/26 (32.5%)	0.897	0.344
Surgical segments, *n* (%)			1.582	0.208
<3 segments	67 (88.2%)	64 (80.0%)		
≥3 segments	9 (11.8%)	16 (20.0%)		
ASA classification, *n* (%)	68 (89.5%)	75 (93.8%)	1.175	0.556
I/II		
III/IV	8 (10.5%)	5 (6.2%)
Preoperative VAS score, mean ± SD	6.1 ± 1.2	6.3 ± 1.1	1.085	0.280

ASA = American Society of Anesthesiologists; VAS = Visual Analog Scale.

*Indicates statistical significance at *p* < 0.05.

### 3.2 Comparison of postoperative gastrointestinal function between the two groups

As shown in Table 2, for the time to first flatus, time to first defecation, and time to first solid food intake, the observation group had significantly shorter durations compared to the control group, with all differences reaching statistical significance (*P* < 0.001). Regarding the incidence of postoperative nausea and vomiting, although the rate in the observation group (5.00%) was lower than that in the control group (14.47%), the difference between the two groups was not statistically significant (χ^2^ = 2.105, *P* = 0.147).

**TABLE 2:** Comparison of postoperative gastrointestinal function between the two groups.

Group	Time to first flatus (h, x ± s)	95% CI	Time to first defecation (h, x ± s)	95% CI	Time to first solid food intake (h, x ± s)	95% CI	Incidence of postoperative nausea and vomiting [*n* (%)]
Control group (*n* = 76)	12.56 ± 4.12	(11.63, 13.49)	22.89 ± 7.23	(21.05, 24.73)	13.58 ± 3.56	(12.43, 14.73)	11 (14.47)
Observation group (*n* = 80)	7.89 ± 2.35	(7.38, 8.40)	15.92 ± 5.34	(14.32, 17.52)	8.87 ± 2.42	(8.02, 9.72)	4 (5.00)
t/χ^2^	8.214		6.785		9.123		2.105
*P*-value	<0.001		<0.001		<0.001		0.147

### 3.3 Comparison of postoperative recovery conditions between the two groups

**FIGURE 1:**
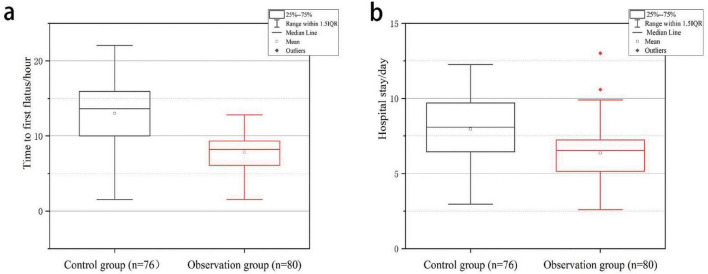
Comparison of primary outcomes between the control group and observation group. **(a)** Time to first flatus (hours) in patients. **(b)** Hospital stay duration (days) in patients.

As shown in Table 3, the resting and moving VAS scores of the observation group were significantly lower than those of the control group, suggesting that the ERAS - based perioperative nursing intervention effectively relieved postoperative pain in patients (both *P* < 0.001). Moreover, the observation group had a significantly shorter urinary catheter removal time, first ambulation time, and hospital stay compared with the control group (*P* < 0.001 for urinary catheter removal time and first ambulation time; *P* = 0.002 for hospital stay) (Figure 1).

**TABLE 3:** Comparison of postoperative pain intensity, urinary catheter removal time, first ambulation time, and hospital stay between the two groups.

Group	Resting VAS score (points, x ± s)	95% CI	Moving VAS score (points, x ± s)	95% CI	Urinary catheter removal time (h, x ± s)	95% CI	First ambulation time (d, x ± s)	95% CI	Hospital stay (d, x ± s)	95% CI
Control group (*n* = 76)	1.23 ± 1.05	(1.00, 1.46)	1.56 ± 1.21	(1.30, 1.82)	14.25 ± 3.72	(13.46, 15.04)	15.34 ± 4.18	(14.40, 16.28)	8.12 ± 2.15	(7.63, 8.61)
Observation group (*n* = 80)	0.56 ± 0.62	(0.42, 0.70)	0.78 ± 0.83	(0.60, 0.96)	8.36 ± 2.01	(7.91, 8.81)	9.27 ± 2.34	(8.75, 9.79)	6.35 ± 1.89	(5.92, 6.78)
t	4.892		5.345		11.567		12.456		3.876	
*P*	<0.001		<0.001		<0.001		<0.001		0.002	

### 3.4 Follow - up findings

A total of 156 patients who underwent ACDF were included in the study, with 80 cases in the observation group and 76 cases in the control group. All patients were followed up, and the average follow - up time was 4.38 (ranging from 3 to 6) months. The relevant data are shown in Table 4.

**TABLE 4:** Comparison of JOA scores and NDI indices at different follow - up time points between the two groups.

Group	*n*	JOA score				NDI index (%)			
		24 h post - op	95% CI	3 months post - op	95% CI	24 h post - op	95% CI	3 months post - op	95% CI
Observation group	80	14.25 ± 0.40	(14.17, 14.33)	16.12 ± 1.03	(15.90, 16.34)	14.23 ± 2.77	(13.61, 14.85)	8.96 ± 1.32	(8.66, 9.26)
Control group	76	12.29 ± 1.24	(11.99, 12.59)	15.33 ± 0.98	(15.10, 15.56)	17.15 ± 2.13	(16.64, 17.66)	10.15 ± 0.60	(9.99, 10.31)
t		5.983		3.214		7.235		3.112	
*P*		<0.05		<0.05		<0.05			

During the follow - up period, no severe complications such as dural perforation, cerebrospinal fluid leakage, or esophageal perforation occurred in all patients. In the observation group, 2 cases of mild dysphagia occurred after surgery, manifested as swallowing pain. In the control group, 2 cases of mild dysphagia occurred after surgery, manifested as swallowing pain and cough; 1 case had hoarseness after surgery. The symptoms of the above 5 cases were improved within 3–7 days after surgery and finally disappeared. Additionally, 1 case in the control group had superficial incision infection after surgery, and the incision healed normally after oral antibiotic treatment. There was no statistically significant difference in the incidence of postoperative complications between the observation group and the control group (*P* = 0.221). The JOA scores and NDI indices of the observation group at 24 h and 3 months after surgery were better than those of the control group, and the differences were statistically significant (*P* < 0.05).

## 4 Discussion

This study assessed the impact of an ERAS-based perioperative nursing pathway on patients undergoing ACDF, focusing on postoperative recovery, functional outcomes, and complications. The findings show that ERAS-based interventions effectively enhance postoperative recovery, optimize gastrointestinal function, reduce pain, and improve long-term functional outcomes, while maintaining a safety profile comparable to conventional care.

Our finding confirm ERAS’s benefits in accelerating gastrointestinal recovery and reducing pain, consistent with lumbar fusion studies. Preoperative nutritional screening and tailored dietary progression likely stimulated gastrointestinal motility and minimized catabolic stress (16). Although the incidence of postoperative nausea/vomiting (PONV) trended lower in the observation group, the difference was not statistically significant, which may reflect the prophylactic antiemetic regimen (ondansetron + dexamethasone) used in both groups (16).

Enhanced recovery after surgery-guided care also reduced resting and dynamic VAS scores, attributed to multimodal analgesia and early mobilization. By minimizing opioid reliance and prioritizing non-opioid analgesics, the protocol reduced opioid-related side effects and enabled earlier ambulation (17). Shorter urinary catheter removal times and earlier ambulation further demonstrate ERAS’ role in accelerating functional independence (18). The shorter hospital stay in the observation group aligns with ERAS goals of streamlining care to reduce costs and improve resource utilization (19). Notably, between-group differences in gastrointestinal recovery persisted after adjustment for operative time, blood loss, and number of levels in sensitivity analyses, suggesting a robust association with the ERAS pathway.

At 24 h and 3 months postoperatively, the observation group had superior JOA scores and NDI indices, reflecting better neurological recovery. Preoperative prehabilitation and postoperative early mobilization likely preserved muscle strength and cervical stability, mitigating postoperative deconditioning (20). These findings support ERAS’ potential to enhance long-term functional outcomes, a critical goal in spinal surgery (21). The overall complication rate was low and comparable between groups, with no severe adverse events. Mild dysphagia and hoarseness resolved spontaneously, while the single case of superficial infection in the control group highlights the importance of early catheter removal and infection-control measures in ERAS protocols (22, 23).

Prior studies have validated ERAS in lumbar fusion surgery, showing reduced hospital stays and improved pain outcomes (13). Our study extends this evidence to cervical spine surgery, where anatomical complexity and higher risks of dysphagia/hoarseness historically justified conservative postoperative management. By adapting ERAS to ACDF–such as via precise hemodynamic control, early mobilization with cervical collar support, and tailored nutrition–we demonstrate that accelerated recovery is feasible and safe in this high-risk population.

The success of our intervention relied on interdisciplinary collaboration (spine surgeons, anesthesiologists, nutritionists, rehabilitation specialists). This aligns with ERAS’ core principle of integrating perioperative care to minimize fragmentation (24).

This study has several limitations. First, it employed a retrospective design, which may introduce bias despite inclusion/exclusion criteria. Second, the sample size (*n* = 156) is modest, and the follow-up period (mean 4.38 months) is relatively short, with no imaging-confirmed fusion status, adjacent segment degeneration, or reoperation data collected. Third, some potentially relevant variables–such as operative time, intraoperative blood loss, smoking status, and diabetes–were not systematically recorded in the retrospective dataset, which limited our ability to fully adjust for confounding factors. Finally, as a single-institution study, generalizability to other healthcare settings is limited. Finally, as a single-institution study, generalizability to other healthcare settings is limited. Future multicenter prospective trials with standardized data collection and longer follow-up are warranted to validate and extend these findings.

## 5 Conclusion

Enhanced recovery after surgery-guided rehabilitation nursing significantly reduces the time to gastrointestinal recovery (e.g., time to first flatus shortened by ∼ 4.7 h), lower pain scores (VAS decreased by ∼1.3 points), and shortens pain management, functional hospital stay by ∼1.8 days in ACDF patients. It also improves functional outcomes, as evidenced by higher JOA scores and lower NDI scores at follow-up, while maintaining a comparable safety profile. By integrating preoperative optimization, intraoperative precision, and postoperative rehabilitation, this approach addresses the unique challenges of cervical spine surgery and advances patient-centered care. These findings support the adoption of ERAS in ACDF protocols. However, given the retrospective and single-center design with short follow-up, prospective multicenter trials are needed to validate long-term outcomes, including fusion rates, adjacent segment degeneration, and quality of life.

## Data Availability

The original contributions presented in this study are included in this article/supplementary material, further inquiries can be directed to the corresponding authors.
